# Informing the development of an E-platform for monitoring wellbeing in schools: involving young people in a co-design process

**DOI:** 10.1186/s40900-020-00219-0

**Published:** 2020-09-03

**Authors:** Claire Grant, Emily Widnall, Lauren Cross, Emily Simonoff, Johnny Downs

**Affiliations:** 1 King’s College London, Department of Child & Adolescent Psychiatry, Institute of Psychology, Psychiatry and Neuroscience, London, UK; 2 The South London and Maudsley NHS Foundation Trust Biomedical Research Centre (SLaM BRC), London, UK

**Keywords:** Adolescent, Mobile health, Smartphones, Mental health, Mood, Patient and public involvement, Co-design

## Abstract

**Background:**

The use of new technologies and methodologies in young people’s mental health research is needed to allow more frequent and reliable sampling. Mobile applications and e-platforms create exciting potential for the collection of large-scale cohort data, however there are various feasibility and ethical issues to consider. Consultation with young people is needed to inform the research agenda, and ensure these technologies are engaging, useful and safe. This article describes the process of Public and Patient Involvement (PPI) with a sample of young people in London, with the aim of i) informing the development of a mood-monitoring e-platform, and ii) providing feedback and advice for researchers developing web-based technologies in the mental health field.

**Methods:**

A total of 26 young people were consulted across four advisory group co-design sessions. All young people were students enrolled at one of the participating London based sixth form colleges, and voluntarily attended a workshop session. Audio recordings of the sessions were analysed using a thematic analysis framework.

**Results:**

We found that young people were engaged in discussions around mobile health technologies and valued the opportunity to collaborate throughout the early stages of the development process The advisory groups identified key considerations for future web-development work to encourage engagement and prolonged use, including, the promotion of trust and transparency, consideration of accessibility, provision of support, production of engaging and functional design, and acknowledgment of specific contextual influences surrounding young people’s wellbeing.

**Conclusions:**

Involving young people in the development process of e-health technologies contributes to optimising the successful adoption and prolonged usage of new methodologies. The thematic map and informant examples can be used to guide researchers interested in developing web-based technologies in the mental health field and will be directly applicable to the development of a mood-monitoring e-platform.

## Plain English summary

Mental health difficulties are common, impacting on the wellbeing, quality of life and overall health of many individuals. Most adult mental health challenges appear before the age of 18, yet there is still a lack of understanding around the factors influencing their development. Research typically relies on young people to reflect back over long periods, making it difficult to observe small changes in a real time setting. A better understanding of short term changes in symptoms could lead to new insights and inform new treatments. Given that young people are early adopters of new technology, an obvious way forward is to use mobile devices to capture young people’s self-reported mood, wellbeing daily events and experiences as they happen in real-time. Such methods would allow researchers and clinicians to gain a more detailed understanding of daily mood changes as well as how daily experiences can impact on mood and the overall mental health of young people.

In order to develop an engaging mobile product that is useful, it is important to involve the end-users (young people) in development, design and testing processes. This involvement is also useful for exploring the ethical and practical concerns of developing mobile health technologies, particularly in the context of young people. This process forms part of a larger research project which explores the possibility of young people using a mobile mood monitoring application within a school setting. This article describes our Patient and Public Involvement (PPI) work with young people, with the aim of i) informing the development of our mood monitoring application, and ii) providing feedback and advice to researchers developing web-based technologies for young people in the mental health field.

We found that young people were engaged in discussions around mobile health technologies and valued the opportunity to collaborate throughout the early stages of a development process. Participants actively collaborated through co-design practises and offered insight into design and function expectations and preferences. The advisory groups provided important context to our project, informing us of attitudes towards commercially available wellbeing applications, as well as mental health school-based research more broadly. In addition, young people highlighted key considerations for future web-development work, including, the promotion of trust and transparency, consideration of accessibility, provision of support, production of engaging and functional design, and an understanding of young people’s specific context.

## Background

Mental health problems in children and adolescents are common, with around 1 in 7 young people in the UK presenting with a diagnosable disorder [1]. Such challenges impact on many aspects of an individual’s life, including social relationships and educational attainment [2], and place exceptional demands on supporting networks and services [3] with 3% of children nationwide referred to specialised Children and Adolescent Mental Health Services (CAMHS) [4]. Early identification and intervention is salient as most adult mental health difficulties present before the age of 18 [5], and the onset of mental health difficulties in adolescence is associated with poorer mental health, physical health, social and economic outcomes in later life [6, 7]. While identification and treatment of child and adolescent psychopathology has advanced in recent years, targeted interventions are limited by lack of varied understanding around disorder development and persistence [8]. There is a need to move beyond the identification of risk factors and begin to understand the nuances influencing varying individual health outcomes in young people [9]. Capturing temporal fluctuations in adolescent mood, and individual exposure to life stressors would help inform future preventative and targeted interventional development [10]. Furthermore, with schools becoming increasingly involved in the detection and support of mental ill-health, there is an additional need for researchers and clinical services to support routine collection of health data at school level [11]. A recent green paper from the British government has highlighted the importance of collaborative working between schools and local services to support the development of resilient and healthy pupils [10], however there are concerns around burden on school staff screening, identifying and supporting student mental health difficulties [11]. As such, there is appetite to develop more effective methods for collecting and interpreting cohort data to facilitate informed interventions for the benefit of schools, researchers and clinical services [12].

Epidemiological approaches to school-based mental health studies can often heavily rely on young people’s retrospective recall due to infrequent school visits for sampling [13]. This limits the ability to investigate timings and granularity of wellbeing reports and impacts on the robustness of representational cohort data. Furthermore, as well as being time and labour intensive [14], these approaches often fail to capture harder to reach students who may be absent from school or unlikely to engage – including children with mental health disorders [15]. A potential solution is to incorporate technological advancements in remote self-reporting in routine school-based data collection [16]. Young people are avid internet and mobile device users and early adopters of new technology, offering the potential for e-platform sampling methodologies [17]. Such approaches have been employed in experiential sampling methods (ESM) for symptom reporting in clinical trials and have been shown to aid the collection of in-real-time data [18, 19]. The promotion of an e-platform for monitoring wellbeing in schools could alleviate burden on staff and have the potential to increase the frequency of engagement from young people [20]. In addition, creating a system to align the collection of data would help to promote consistency between school, health care and research outcome measures [21]. As well as routine data collection, a mental health e-platform has the potential to facilitate health promotion and intervention programmes, provide up-to-date signposting information and act as a recruitment mechanism into health research [22].

While such digital technologies create exciting potential, careful consideration must be made to the ethical, practical and potential exclusionary implications of this advancement [23, 24]. Consulting with young persons’ advisory groups (YPAGs) is essential for understanding specific barriers to engagement, including digital access and competencies, and for exploring key concerns, practicalities and expectations of this health technology. In addition to public and patient involvement (PPI) becoming increasingly valued as a component of research acceptability and feasibility, there is also a legal obligation under Article 12 of the United Nations Convention on the Rights of the Child (UNCRC) to give children the right to have their views given due weight in all matters affecting them [25, 26]. While forms of PPI are increasingly prevalent in health research, there are concerns that the power dynamic between ‘research community’ and ‘public’ limits the impact of meaningful and effective collaboration [27]. To address such concerns, previous adolescent e-health research has employed a collaborative co-design approach with young people throughout web-development processes [28, 29]. There are various roles a young person can occupy in a co-design collaboration, including a user, tester, informant or design partner [30]. In the role of informant, young people contribute to the design process through offering insight and feedback on existing technologies, or on prototypes/early design ideas. Once the technology is developed, children may again offer input and feedback [31]. This cyclical framework informing the development and direction of digital tools has been thought to increase feelings of ownership and levels of engagement with participants [32]. Furthermore, such an iterative process allows researchers to refine the clinical responsibility and level of interaction which have been identified as key challenges within e-health technologies [33]. In order to optimise the potential of an e-platform, the expectations and motivations of young people must be understood. This PPI project describes a co-design process with young people acting as informants and contributes to a larger body of work (Medical Research Council (MRC) Pathfinder Grant MC_PC_17214), assessing the acceptability and feasibility of embedding a mood monitoring e-platform into schools for students aged 16+. The overall project aims to understand the ethical issues surrounding young people’s e-cohorts, explore the feasibility of online recruitment mechanisms (including meaningful ways to involve young people), and gain information on factors influencing participant retention. This PPI focuses on exploring i) young people’s thoughts on mental health; ii) features and functions of an e-platform for monitoring wellbeing; and iii) barriers, including ethical and safeguarding concerns, to engaging with e-health technologies.

## Methods

### Aim

Our aim was to gather voices from young people (aged 16–18) attending a secondary school or sixth form in London. Advisory groups set out to explore initial thoughts and concerns around; i) young people’s mental health; ii) features and functions of existing e-platforms for wellbeing monitoring; and iii) barriers to engaging with e-health technologies.

### Design

The discussion guide for the young persons’ advisory group (YPAG) session was designed to be interactive and inclusive. As outlined in co-design literature, low-tech materials, interviews and design feedback on existing platforms or prototypes can all be used as methods for young people acting as informants in a co-design process. The sessions were designed to incorporate this multifaceted approach, and included task-based exercises, group discussion and feedback presentations. The final format was reviewed by colleagues with clinical expertise in Child and Adolescent Mental Health Services (CAMHS) and school-based mental health research at the National Institute for Health Research (NIHR) Maudsley Biomedical Research Centre (BRC). A mock advisory group session was held with a selection of the above colleagues and feedback was given on the content, length and format of the meeting. Furthermore, all materials (including adverting leaflets, discussion guides and supplementary resources) were circulated to clinical academics in CAMHS for comment.

To encourage collaboration with the project and ownership over contributions, participants were encouraged to identify, organise and offer insight into patterns of meaning within the group discussion. This was facilitated through visual grouping tasks and feedback from group members [34]. Firstly, a scenario was presented to the YPAGs, describing a young person who had been feeling increasingly anxious and was having difficulty sleeping. Using visual prompts of a wall and ladder, participants were asked to write down perceived barriers for accessing support, as well as opportunities to enable help-seeking. Participants were invited to present their thoughts back to the group, and common themes were identified and discussed. Following this, young people were asked to write down words or phrases they associated with ‘mental health’ and assisted in grouping these into categories. To gain specific feedback on existing eHealth technologies, screenshots of commercially available and research specific mood monitoring apps were displayed in a gallery format. The YPAG was invited to comment anonymously by annotating the screenshots with likes and dislikes of the design and content. In the two final groups, a blank phone template was provided to give an opportunity for young people to sketch ideas for app features and functions. This section was designed to allow informants to determine the direction of web development processes, and so feedback around specific products (e.g. embedded commercial apps, research web-questionnaires) was essential. Finally, a discussion was had around safety and security, including privacy concerns, use of data and safeguarding procedures. Throughout all YPAG sessions, notepads were provided for young people that did not want to contribute to discussions in person. Furthermore, contact details of the research team were given for any follow up questions/comments, and a debrief leaflet was provided with signposting information to relevant young people’s mental health services and helplines To ensure young people were actively playing the role of informants, YPAG facilitators allowed the discussion to be led by participants and aimed to listen to group discussion amongst young people. While pre-set prompts and activities were used as a guide, the young people had control over how these were interpreted.

### Participants

An established convenience sampling method [35] utilising existing connections with KCL was used to recruit schools to participate in the co-design process. After initial engagement meetings with several schools, two diverse schools (see Table 1) had capacity to take on the project. Participants were recruited through two secondary schools in London with varying profiles according to publicly available government data. All students aged 16+ attending participating schools were invited to take part in sessions through advertising leaflets disseminated by registration teachers. The age range of 16+ was set in line with the scope of the larger feasibility project.

**Table 1:** Participating school characteristics

	School A	School B	National average
Type	Secondary with Sixth form	Secondary with Sixth form	–
Selective	Non-selective	Non-selective	–
Size	2070	1133	–
PROGRESS 8 score	0.51	0.1	−0.03
Special Educational Need (%)	8.1	8.9	10.8
FREE SCHOOL MEALS (%)	19.6	61.8	27.7
English NOT FIRST language (%)	23.4	49.7	16.9

The final sample consisted of 26 young people in Year 12 across four advisory group sessions (*n* = 6, n = 6, *n* = 7, n = 7). No individual demographic information was collected to ensure anonymity. Sample size was ascertained through an iterative research process and recruitment stopped at the point of data saturation [36]. These numbers were in keeping with recommendations in previous qualitative group research [37].

### Analysis strategy

Despite increasing interest around involving PPI groups as an integral part of healthcare research, there are limited resources exploring effective and rigorous methodologies for analysing and interpreting collected data [27]. In order to intertwine YPAG findings with project development and theoretical understanding, a multidimensional analysis strategy was considered. A thematic framework was employed to allow researchers and young people to understand patterns of meaning within the collected feedback [38]. Feedback from participants were explored, and audio recordings from the YPAG sessions were listened to (independently by CG, EW and LC), to ensure familiarisation of data and to recapitulate content. Initial codes were collaboratively produced and refined, before more conceptual themes were developed through further discussion. The final themes were disseminated to the participating young people for comment prior to formalising findings.

## Results

The five resulting themes from across the YPAG sessions are summarised below.

### Theme 1. Perception of young People’s mental health

Throughout the sessions, group members discussed the importance of understanding the specific needs and context surrounding young people’s mental health. Participants wanted health related issues to be taken seriously, validated and respected by supporting adults. There were concerns around disclosing information and being dismissed.

“We should always believe when someone says something to us, like confides in us. You just don’t know what they’re going through.” (G2).

There were additional comments around the desire for non-judgemental support and advice around risky health behaviours that influence on mental and physical health (such as alcohol/drug consumption, sexual activities and excessive social media use).

“Teachers always blame it on you though, like, if you feel bad because you’ve been smoking or drinking, they’ll just be like that’s because of you.” (G1).

Members also felt positivity towards embracing new technology in mental health and wellbeing measurement, however commented on the potential reluctance to adopt these methodologies in some populations.

“The older generation always say phones are making us all have mental health issues, and I think it maybe has impacted us in some ways … but I think it can also be a way to help.” (G3).

Group members also recognised a spectrum of mental health and wellbeing needs.

“If you look back, mental health support and stuff has come a long way and I think people are beginning to realise that we all have wellbeing needs” (G1).

The group also discussed the need for supporting networks to understand life stressors specific to contemporary adolescents when identifying and treating wellbeing needs.

“I mean life can be stressful with like exams and school and social media pressures and stuff.” (G4).

### Theme 2. *Trust*

The YPAGs discussed the importance of transparency and protection of young people’s data and its use - including willingness to share data, anonymity and fear of data misuse.

“There a feeling of ‘what are you doing with our data’, because lots of places give it to like third parties. I don’t want to have my life recorded if I don’t know what’s going to happen with that information.” (G3).

Group members agreed that clear and transparent data policies would make individuals more likely to use and trust an e-platform.

“As long as they tell you like honestly what they are doing with your information, I wouldn’t mind sharing with researchers.” (G4).

Young people felt it was important to trust the team behind the web development and expressed scepticism around corporate companies’ intentions. Group members felt that an e-platform would be legitimised if it was promoted through a trusted source, such as school or a health care provider.

“I want to use something that was built by people that care, like actual humans, not just companies trying to get money and data from you. It would be good if teachers could suggest it to students.” (G1).

It was felt that flexibility should be incorporated into the data policies to allow individual users to control the use of their data. Most group members reported willingness to share data at group or cohort level but remained reluctant to share identifiable data with schools and researchers.

“If it’s really and truly anonymous, then under no circumstances should that be broken.” (G2).

### Theme 3. Accessibility

The participants felt that any platform developed should optimise accessibility to all students. During the group sessions, general barriers and enables of help seeking behaviour were discussed (including factors for accessing e-support). It was felt that the stigma of mental health influenced individual help seeking, as well as the choice of mobile phone application or website accessed.

“The title of it needs to be subtle, because if it’s literally something like ‘mental health help’, then they are going to try and hide it and won’t want it on their phone.” (G2).

Furthermore, individual differences and issues around diversity and inclusion were discussed in relation to accessing and adhering to health programmes.

“There’s definitely issues with toxic masculinity … Plus it’s harder to access therapy and stuff if your family don’t talk about this or maybe just can’t afford it.” (G1).

“If your parents don’t necessarily know what you’re going through, you can’t really go up to them and get them to pay a £15-a-month subscription.” (G4).

The groups highlighted that the ease of accessing (i.e. downloading, signing up to and beginning to use) an e-platform would be an important factor for individuals showing initial interest. This also included practical issues with data usage, storage capacity and battery demand of the platform. The participants also discussed the accessibility of content on the platform, with developers ensuring that the level of information is appropriate for the age category intended.

“Don’t put the information in a long, boring agreement thing because nobody reads that.” (G3).

### Theme 4. *Support*

The groups felt that an effective and engaging e-platform would provide an appropriate level of information and support to facilitate positive mental health and wellbeing. Participants discussed the ability for young people to engage in self-help, and frustrations around patronising approaches to mental health support.

“I think teachers and parents can sometimes not give us enough credit of being able to deal with things on our own … like we are more independent at this age and I think we should be able to have access to information that we can use to help ourselves without necessarily adults being involved.” (G3).

However young people also acknowledged the variety of support needed for each user and highlighted the need to provide emergency responses when needed.

“I think there needs to be a kind of safeguarding system for when people are in danger or might hurt themselves.” (G4).

The groups also discussed the current lack of wellbeing support for young people who don’t present to mental health services or have a clinical diagnosis.

“It feels like you either have to wait to get to the point of being so bad that you’re on medication, or it’s no input and you can just deal with it yourself” (G3).

Young people identified choice in mental health support was important, and an e-platform should provide options in the services and self-help available to give individuals ownership in their help seeking.

“It’s best to give the information on services available and give people the choice of whether to access them or not.” (G1).

### Theme 5. *Design*

Young people stressed the importance of design in the acceptability and feasibility of a mental health e-platform. Participants discussed design preferences with reference to existing mental health phone applications and websites known to them.

“It needs to be designed well. If it looks like it’s made in 2000, I’m not using it … I mean I will literally think ‘can I really trust it if it looks like that.’” (G1).

Furthermore, the layout and presentation of questions was thought to be an important factor in young people’s responses and level of engagement.

“We don’t like being boxed and like categorised, maybe even something like a slider so you don’t have to say, ‘I’m this’ or ‘I’m that’.” (G3).

Having the ability to customise and personalise profile settings was discussed as a favourable feature in an e-platform. This included comments on personalised colour palettes, icons and notification reminders. Individuals also expressed an interest in having a feedback function for reviewing entries.

“People want to get results. if you’re putting in information you want an answer to that. So, I guess it’s helpful to get some kind of feedback in that way.” (G2).

### Example co-design informant input

**Table:** 

Theme	Request	Impact on e-platform
Young People’s Mental Health	Acknowledge the specific contexts which young people face mental health challenges.	When documenting life-events, the e-platform presents the user with categories, including school, to contextualise their experiences.
	Allow autonomy in help-seeking and health management.	The platform provides feedback graphs to the user to allow for reflection on mood variation across time and place. It also provides help-seeking resources.
	Minimise the focus on ‘mental health’ problems.	The title and icon of the app are not associated with mental health. Questions asked are addressing wellbeing and life-events rather than clinical outcome measures.
Trust	Provide a space to document thoughts and feelings without fear of information being shared.	The platform promises anonymity to the user. The only instances in which this is broken, is if the user was in immediate danger to themselves or others.
	Clear and concise information on how the data is handled.	The platform has a data privacy policy which is written in lay English terms to allow for complete transparency over the sharing and storing of data. Data sharing preferences are also made flexible, with control given to the user and their account.
	Ability to contact the web-developers and others involved.	Contact information for the web-development company and researchers involved are provided to all users, and feedback on app stores regularly checked for queries.
Accessibility	Inclusivity in every character used.	The platform uses an avatar character which is inclusive of race, age and gender.
	Do not charge for app or in-app features.	The platform is a free service provided for schools, no individual user or school will be charged to access its features.
	Make information understandable and digestible.	The platform has tried to minimise ‘words per page’ and has provided a break-down of information on the research which is accessible at all times in an embedded PDF document.
Support	Having a clear safeguarding procedure for at-risk users.	The platform has clinical advisors that will assist in risk screening of any inputting of free-text boxes.
	Offering support services for those in need.	Embedded as part of the app, there is a ‘support’ tab which lists a range of local and national support resources. Hyperlinks to webpages, phone numbers and app downloads are built in.
	Transparency of therapeutic ability and scope.	As part of the sign-up process, the platform outlines the limits of its therapeutic ability – including the frequency of risk screening and the lack of immediate support for users.
Design	Do not box responses into categories.	Most responses on the app are collected through a ‘sliding scale’ to offer flexibility. In sections where this is not possible, an ‘other’ option or free-text box is provided.
	Avoid using triggering colours.	The app does not use ‘red’ or ‘green’ colours to reflect ‘good’ and ‘bad’. The colour scheme is pastel, and the avatar character is purple, which users felt was gender neutral.
	Flexibility in the nature and frequency of notifications.	The app allows the user to determine when notifications are set, with setting preferences at the sign-up process as well as complete flexibility to change this in user settings throughout engagement.

## Discussion

This study provided an opportunity to explore key features of a successful e-platform for monitoring mental health and wellbeing within a school setting for young people 16+. Findings support some existing literature, highlighting the importance of design and customisation on e-platform uptake and retention [29], and provides novel insight into young people’s expectations and preferences for digital health. Very few studies have reported the process of co-design with young people in wellbeing digital technologies. The exploratory nature of this PPI contributes to understanding the needs of young people, specifically the functions required in a mood monitoring e-platform and the process of co-design. This programme of work provided an insight into young people’s perceptions of mental health, their ideas and concerns surrounding eHealth technologies and ideas around what an effective eHealth platform may look like.

The paper describes the facilitation of 4 YPAG sessions and provides an overview of five key considerations for the development of a mental health e-platform, with each broad theme incorporating more specific requests and expectations from young people. Conversations with participants highlighted the importance of understanding the specific needs and context of young people’s mental health throughout the research process, and in relation to the development of an e-platform. Across all groups, participants identified frustration towards frequent dismissal of mental health difficulties from adults, and highlighted factors (including social media pressures, exam stress and risky health behaviours) that feature as part of young people’s everyday lives. While the sessions promoted interesting discussions around the particular context of participating students, future research should seek to consult with their population of users.

In addition, young people felt that trust was important in the acceptability of any e-health technology, in terms of the legitimacy of the developers, the transparency of data management and the promise of confidentiality. Future e-health research should consider ways in which to translate the complexity of data policies into accessible language to ensure participants are fully informed of their rights and options. Young people also identified additional accessibility considerations, such as the promotion of diversity and inclusion. It was highlighted that the acknowledgement of specific barriers and enablers to help-seeking behaviour and programme engagement from young people would act as an important feature in product uptake and acceptability. Furthermore, the sessions called attention to the need for support provision to promote positive mental health and wellbeing in a mental health e-platform. Young people felt that self-help materials and up-to-date signposting information would be sufficient for the majority of users, however identified the need for safeguarding protocols for individuals in more immediate need. Finally, the groups emphasised the importance of design, and identified preferred features and functions for user interface. Participating young people expected high standards for web-based usability and design which should be considered in future health care provision and research methodologies. The YPAG participants were able to critically evaluate commercially available health platforms, demonstrating the expected standards of e-health products. Future research should continue to harness the expertise and preferences of digital natives. These findings could be used as a resource for future research into adolescent mental health, by providing a set of reoccurring themes in e-health acceptability and feasibility.

Findings from this study compliment other PPI work aiming to co-design with young people and reiterate the need for a product to be accessible, easy to use and well designed. However, there seem to be nuances in the expectation of users depending on who the population are, and what the primary purpose of the app is. For example, while previous PPI with mental health service users has highlighted the importance of clinical responsiveness and ‘outreach’ approaches within apps [29], these findings suggest that within non-clinical populations, it is the transparency of clinical expectations that is paramount [33]. Understanding the ways to make mood-monitoring apps that provide an appropriate level of support for the users further exemplifies the need for flexibility and user-centred design [31].

### Impact of advisory groups

Measuring impact was not the primary aim due to the iterative nature of this PPI and subsequent challenges in obtaining quantifiable measurements on impact. However, reflecting on the qualitative feedback from participants, the YPAGs were considered to be a useful tool in assessing acceptability and feasibility of an e-platform, informing the web development process, and increasing transparency in ongoing mental health research. Reference was made to what Brett et al. describe as ‘impacts on users’ [39], particularly in terms of personal benefits, for example users sense of empowerment and feeling listened to. The participants also expressed interest in taking part in future YPAG sessions, suggesting young people enjoyed being involved in the research process of their personal involvement. Continuing a dialogue between researchers and school students could increase interest and awareness of health and epidemiological research, a consideration to be made when measuring the impact of future PPI work on recruitment and attrition.

### Reflections and limitations

The evolving nature of this PPI work allowed for ongoing improvement in the way that the sessions were conducted. Such anecdotal findings may be useful for future PPI work involving young people. The research team agreed that while it was logistically easier to host sessions within school times/premises, it was important to try and dissociate the session with the school itself. For example, it seemed that young people were more likely to speak openly when members of staff were not present. It was also felt that due to the limited time with the YPAGs, having existing relationships with and within the group was helpful for establishing rapport. For example, in groups where members all consisted of the same registration or tutorial class, young people were generally more responsive to group tasks. This was most apparent when participants chose to reflect on personal mental health difficulties or experiences with eHealth technologies. While there were no expectations or obligations for young people to do this, in groups where students perceived a trusting environment, it did lead to a more in-depth discussion into the real-life application of eHealth technologies.

There are several limitations to this PPI project. Firstly, while efforts were made to be inclusive throughout the YPAG recruitment process, the convenience sampling methods used did not guarantee representational participation. This is a challenge with many PPI initiatives as the voluntary nature and small sample sizes of the work tends to fail to capture harder-to-reach populations and to ensure diversity. It should also be noted that this PPI was London-based, and findings therefore may be specific to the viewpoints of students attending such inner-city London schools. While the findings offer important local insight into mental health and new technologies, the limited geographical scope of the work should be acknowledged and findings can therefore not be generalised more widely. Similarly, while the age range in this PPI is based on the intended audience for the mood monitoring app, it does limit the findings applicability to younger audiences (i.e. under the age of 16). In addition, no formal measures were used to quantify the impact of the PPI on the project. An impact evaluation methodology would need to be embedded early on in order to effectively demonstrate impact. Future research should consider the ways in which the impact of young people’s voices can be formally evaluated.

Furthermore, while each session was intended to act as a cyclical workshop, with ongoing feedback and iterations to the web-development proposal, there were limitations in the implementation of the ‘co-design’ approach. Each YPAG was consulted on one standalone occasion and this was predominantly due to the restrictions of exam schedules and school timetables. It would have been interesting to collaborate with the same YPAG members throughout the process of the web development. However, the method described did allow for a greater number of voices to be heard overall and prevented bias towards the product as a result of personal investment in the process.

## Conclusions

An e-platform delivered via schools presents an opportunity to unobtrusively capture a significant amount of data from young people in everyday life. This data allows opportunity to improve the detection, prediction and understanding of mental health difficulties among young people. Whilst still an emerging area of research, we found that in principle, there was a good amount of support from young people for this type of research and we found it was feasible to involve young people and use their insights to shape the developing of an e-platform for mental health research.

Involving young people in the development process of e-health technologies offer unique insight for ensuring the successful adoption and usage of new methodologies. This current PPI project outlines five key themes to consider when developing e-health technologies: perception of young people’s mental health, trust, accessibility, support and design. Insights from advisory group work can be used to inform researchers interested developing web-based technologies in the mental health field and will be directly applicable to the development of adolescent mood-monitoring e-platforms.

This project provides guidance for the development of a mood-monitoring e-platform, and forms part of a larger body of work. The e-platform developed from this project aims to be piloted in at least one secondary school this year (King’s College London Ethics Reference: MOD-19/20–13,071). Further publications will aim to describe the process of embedding this platform as part of a school system and offer insight into student acceptability and adherence through further PPI and app usage metrics. All schools that engaged with this piece of PPI will be invited to take part in a whole-year or whole-school roll out of the app.

## Data Availability

Not applicable.

## Abbreviations

YPAG: Young Persons’ Advisory Group
CAMHS: Child and Adolescent Mental Health Service
MRC: Medical Research Council
NIHR: National Institute for Health Research
PPI: Patient and Public Involvement
ESM: Experiential Sampling Methodology
G: Group
